# Notes on Syphilis and Dermatology

**Published:** 1874-03-15

**Authors:** 


### NOTES ON SYPHILIS AND DERMATOLOGY.

*Translated for* THE EXAMINER, *from La France Medicale of Dec. 20th*, 1873, *Jan. 3d*, '74; *Le Progres Medical, Dec. 27th*, 1873.

GUMMY TUMOR OF THE RIGHT INDEX (*La France Medicale*).—Dr. P. Labarthe was consulted by a cabinet-maker, who exhibited to him a tumor of the size and appearance of a pigeon's egg, situated upon the radial side of the first phalanx of the index finger; painless; well-defined; having a smooth surface, resting upon an indurated base, with a central portion somewhat softer, but non-fluctuating, and presenting an uniformly reddish-violet tint.

It had existed for the previous three months and a half, and had been poulticed with subsequent application of compresses, to no purpose.

The slow development of the growth, its painlessness, and the absence of fluctuation, pointed to something distinct from the enlarged bursæ which form in this location as a consequence of the manipulations of artisans.

The history of the case precluded the possibility of the previous entrance of a fragment of some foreign substance, introduced through the skin, and becoming subsequently encysted. These occurrences are, besides, exceedingly rare. There was no history of rheumatism.

Subsequent to this interview, Dr. Labarthe read an article by M. Verneuil, in the *Gazette Hebdomadaire*, on "Tertiary Syphilitic Affections of the Subcutaneous and Tendinous Bursæ," in which two cases were detailed. In the first, a tumor developed, in 1872, from the bursa serosa, situated upon the anterior tuberosity of the tibia, in a woman of fifty-two, who had an infecting chancre in 1863. In the second case, that of a young physician, affected with the disease four years before, the same development originated in the bursæ of the muscles of the thighs.

Dr. Labarthe at once sought his patient, and discovered, on questioning him, that he had suffered from a blennorrhagia when seventeen years old; and three years later had, consecutively, infecting sore, crusts upon the hairy scalp, and soreness of mouth and fauces. The diagnosis of a syphilitic gummy tumor was at once concluded upon, and became justified by the result, as the tumor disappeared completely in one month, after local frictions with mercurial ointment, and the internal exhibition of the iodide of potassium.

Retro-Pharyngeal Adenitis (*La France Medicale*).—A fleshy woman, twenty-two years of age, of sanguine temperament, eight months pregnant, and syphilitic since her conception, consulted M. Depres for sore thoat and inguinal adenopathy. He established, mucous patches of the velum and palate, and a tumefaction as large as a nut, on the posterior and left lateral pharyngeal wall, surrouded by granulations. By the finger, it was ascertained that the swelling was occasioned by hypertrophy and inflammation of the lymphatic ganglia of that region. The indurated mass was distinctly rounded, almond-shaped; painful on pressure; and somewhat mobile. Another similar mass, smaller, and only as large as a pea, was distinguished below. Otherwise the subjective symptoms were not marked—there was neither dysphagia, fever, nor affection of the auditory apparatus; merely a somewhat painful motion of the jaws. There was no nasal voice, fluctuation, nor the existence of an œdematous zone around the induration; hence resolution was expected. It was regarded as a case of benign syphilis, the mucous patches of the throat and vulua, and the pharyngeal and inguinal adenopathy being its sole manifestations.

The patient was entirely relieved by the usual internal treatment, in combination with an ioduretted solution of zinc, applied locally.

Syphilitic Cirrhosis of the Liver (*Le Progres Medical*).—A woman who was profoundly cachetic, and who had never suffered from paludal fevers, nor the sequelæ of alcoholism, had required several courses of mercurial treatment for the relief of a complete series of secondary and tertiary syphilitic accidents: notably periostitis and incomplete amaurosis, from probable choroiditis. The malady had existed for some three or four years, and had brought about various digestive disorders and general feebleness. She died soon after her removal to hospital.

At the autopsy, the liver was found to weigh but little more than one pound, and to measure, in its transverse diameter, seven and one-half inches. Its aspect was exceedingly characteristic. It exhibited a series of irregular prominences and depressions on the superior and inferior surfaces. At the level of the depressions, its fibrous envelope was thickened and whitish, but did not appear to extend by deep prolongations into the hepatic parenchyma. In the intervals of these fibrous tracts, the entire surface of the organ displayed a considerable number of small yellow granulations, as large as a mustard-seed. The vena porta was permeable, and the subhepatic veins returned an injection passed into the former, while the intralobular veins on section exhibited the coloring matter introduced. The microscopic examination of the liver revealed islets of cellules, separated by a strip of yellow connective tissue. There was evident proliferation of connective tissue about the branches of the portal vein. Syphilitic cirrhosis was determined to be the cause of death.

In the discussion of this case before the Anatomical Society, M. Lucas-Championniere stated that the microscopical characters of the specimen exhibited, were not demonstrative of syphilis, since many alcoholic cirrhoses possessed similar appearances. M. Cornil remarked that the liver was small, lobulated, and irregular. These are the characteristics of every cirrhosis arrived at the atrophic condition, whatever be its origin. We cannot, therefore, decide that this woman was syphilitic. The most interesting feature of the specimen is the injection of the still permeable capillaries, despite the ascitic symptoms observed during life. This fact stands in marked contrast with the ideas of the German authors, who admit that, in these cases, the hepatic circulation is dependent solely upon the hepatic artery. Here is undoubedly an interesting field for observation and research.

ERYTHEMA MARGINATUM, AND ITS RELATION TO RHEUMATISM (*Le Progres Medical*).—There are two principal varieties of the cutaneous affections designated as erythema. In the one class, the disease depends generally upon an external and a local cause, and is always circumscribed and limited to a single region; in the other, it is the result of an internal cause, and is more or less generalized in extent. Authors are generally agreed as to the fact of some general condition, which is manifested by a generalized erythema. They differ, however, in the definition of this general condition. This is true of erythema nodosum, and also of the varieties erythema papulatum, marginatum, etc., classified by Hebra under the general denomination of polymorphous erythema.

Those who rely upon the co-existence of articular pain with this exanthem, consider it to be a cutaneous manifestation of the rheumatic diathesis. Bazin declares that "Erythema papulatum, erythema marginatum, and erythema nodosum, are arthritic in character." Trousseau, in his "Clinic of the Hotel Dieu," Ferrand, in his "Thesis on the Rheumatic Exanthemata," and Legroux, in the discussion before the Medical Society of the Hospitals, hold to the same opinion.

Others, however, do not consider that the articular pains authorize them to accept this conclusion. MM. See and Vigla, in the Report of the Society referred to above, affirm that these vague sensations of pain in the neighborhood of the joints, or in the continuity of members, are totally different from the rheumatic pains.

"I have seen," says Gubla (*Bulletin de la Societie Medicale des Hopitaux*), "cases of erythema nodosum, in which the articulations were painful, and distended with effusions. I have even seen the same disease complicated by endocardiac souffle, which might lead to suspicions of rheumatic complication; but I believe there was in these cases merely a nodose erythema, with morbid manifestations in the serous linings of the joints and internal membrane of the heart, resembling, but not actually producing, rheumatism. I protest against the application of the term rheumatism to certain painful sensations in the joints. M. Trousseau would have us believe in a scarlatinal rheumatism; and I once told him that if an effusion into a joint was proof positive of rheumatism, our medical philosophy was worthless. The painful sensations of the joints in scarlatina and erythema nodosum are rheumatoid, not rheumatic.

M. Hardy (*Nouv. Dict. de Med. et Chir.*, Art. "Erythema,") expresses himself in a similar way. He has noticed the fact of these articular pains in some cases of erythema papulatum, and, on two occasions, inflammatory affections of the endo and pericardium; "but in the majority of cases, these articular phenomena are wanting. The rheumatismal arthritis is quite similar to that occurring in scarlet fever." He says, further, "Generalized erythematous eruptions depend upon a general cause, and are so similar to the eruptive fevers that they might be arranged in the same class."

Accordingly, in his "Internal Pathology," M. Hardy describes the localized erythemata in connection with the diseases of the skin; and the generalized eruptions are considered in the chapter on Eruptive Fevers.

Jonathan Hutchinson goes a step further, and seems disposed to admit that one attack of erythema nodosum or papulatum, produces immunity from a second, and urges men of observation to consider whether it is not contagious.

Such are the principal opinions now prevalent on the subject of generalized erythematous eruptions. They have been detailed as an introduction to the following case:

A servant girl, previously healthy, in good flesh, twenty-one years of age, and a blonde, entered the Hotel Dieu April 11, 1873. Neither she nor her parents had ever suffered from any form of rheumatic complaint. Three days before, her menstrual secretion had been arrested, after a two days' flow (instead of eight, which had been her usual period of sickness), and she had, since that date, suffered from lassitude, general malaise, and moderate cephalalgia.

On admission, there was established, a few sibilant rales in the chest; a pulse of 100 beats; a slightly red and coated tongue, with some nausea; slight redness of pharynx, and moderate heat of skin. On the surface of the abdomen and back were disseminated spots of variable form and size (circular and elongated and semi-lunar), of a delicate pale rose, almost yellow, color in the center, and moderately red at the edges, the latter tint disappearing temporarily under pressure, while the former persisted, especially in the larger patches. The center seemed somewhat depressed, while the borders of each spot were elevated to such an extent as to be appreciable by the sight and the touch. The eruption had begun upon the arms, where merely brown maculæ could now be detected, and had extended to the surface of the chest by the evening of the 10th, when it assumed a paler and more yellow (almost copper-colored) hue. This character, taken in connection with the form of certain of the patches, made room for a suspicion of syphilis. The eruption was accompanied by no pain nor itching.

The eruption gradually invaded the entire surface of the integument, as far as the lower extremities, becoming rather more yellow in shade, up to the evening of the 13th, when the patient complained of pain in the elbows and knees. This was subsequently accompanied by very great tenderness, and rosiness of the integument covering the joints. The eruption, meantime, persisted, but commenced to decline on the 15th, when a systolic murmur became audible at the cardiac apex, and the first sound of the heart imperfectly audible at its base. On the 17th, the eruption had completely disappeared. Then succeeded abundant night-sweats, friction sounds over the pericardium, several returns of the eruption on the trunk and lower extremities, alternating, apparently, with articular pain and effusion; and finally, distinct aortic insufficiency was displayed to the eye by the sphygmograph. The joints of the digital phalanges became involved somewhat later, and exhibited protuberances (nodosities of Heberden), which were slow to improve.

It was concluded that, at least in certain cases, the exanthems designated as erythema marginatum, papulatum, etc., have an evident connection with the rheumatic diathesis.

